# Blocking PD1/PDL1 Interactions Together with MLN4924 Therapy is a Potential Strategy for Glioma Treatment

**DOI:** 10.4172/1948-5956.1000543

**Published:** 2018-08-06

**Authors:** Natalia Filippova, Xiuhua Yang, Zixiao An, Louis B Nabors, Larisa Pereboeva

**Affiliations:** 1 Department of Neurology, Division of Neuro-oncology, School of Medicine, University of Alabama at Birmingham, Birmingham, USA; 2 Division of Hematology and Oncology, School of Medicine, University of Alabama at Birmingham, Birmingham, USA

**Keywords:** PDL1, HIF, Gliomas, Cancer therapy, MLN4924, Brain tumour, Immune checkpoint

## Abstract

**Objective:**

MLN4924, a pharmacological inhibitor of cullin neddylation, resulted in glioma cell apoptosis, deregulation of the S-phase of DNA synthesis and thus, offers great potential for the treatment of brain tumours. However, targeting the neddylation pathway with an MLN4924 treatment stabilized the hypoxia-inducible factor 1A (HIF1A), which is one of the main transcriptional enhancers of the immune checkpoint molecule PDL1 (programmid death ligand-1) in cancer cells. The influence of immune checkpoint molecules on glioma progression has recently been discovered; PDL1 overexpression in gliomas corresponds to a significant shortening of patient survival and a decrease of the anti-tumour immune response. We hypothesize that i) PDL1 is up-regulated in gliomas after treatment with MLN4924 and induces T-cell energy; ii) co-utilization of the PD1/PDL1 blockage with MLN4924 therapy may reduce T-cell energy and may engage MLN4924-induced tumour disruption with the immune response.

**Methods:**

PDL1 expression and its immunosuppressive role in gliomas, glioma microenvironments, and after treatments with MLN4924 were assessed by utilizing methods of immunohistochemistry, molecular biology, and biochemistry.

**Results:**

We confirmed PDL1 overexpression in clinical brain tumour samples, PDGx and established glioma cell lines, extracellular media from glioma cells, and CSF (cerebrospinal fluid) samples from tumour-bearing mice. Our primary T-cell based assays verified that the up-regulation of PDL1 in tumour cells protects gliomas from T-cell treatment and reduces T-cell activation. We found that a pharmacological inhibitor of cullin neddylation, MLN4924, exhibited strong cytotoxicity towards PDGx and established glioma cell lines, *in vitro*, with an IC50’s range from 0.2 to 3 uM. However, we observed a significant increase of HIF1A and PDL1 in mRNA and protein levels in all glioma cell lines after treatment with MLN4924. The MLN4924-dependent induction of PDL1 in gliomas resulted in T-cell energy, which was blocked by a blockage of the PD1/PDL1 interaction.

**Conclusion:**

We conclude that i) PDL1 up-regulation in gliomas and the glioma microenvironment is an important chemotherapeutic target; ii) MLN4924 therapy, combined with a blockage of the PD1/PDL1 pathway, should be considered as a potential strategy for glioma treatment.

## Introduction

Programmed death ligand-1 (PDL1) is a major immunological checkpoint ligand molecule, and it is up-regulated in tumors and tumor microenvironments of different types of cancer [1-6]. Clinical trials report that the inhibition of PDL1 interaction with programmed cell death receptors (PD1), expressing on various types of immune cells (including antigen presenting cells, effector T cells, natural killer cells (NK), thymocytes, myeloid cells), leads to durable tumor control by the immune system and the suppression of different types of cancer [7-10]. At least twenty-seven clinical trials were initiated to evaluate PDL1/PD1 inhibitors alone or in combination with other drugs for the treatment of brain tumors (including gliomas and GBM) during 2014-2017 period: NCT02550249 (2015), NCT02423343 (2015), NCT02017717 (2014), NCT02311920 (2015), NCT02337491 (2015), NCT023311582 (2015), NCT01952769 (2014), NCT02336165 (2015), NCT01375842 (2011), NCT02829931 (2016), NCT02313272 (2014), NCT02798406 (2016), NCT03058289 (2017), NCT02335918 (2015), NCT02852655 (2016), NCT02526017 (2015), NCT03233152 (2017), NCT02968940 (2016), NCT02327078 (2014), NCT02794883 (2016), NCT02311582 (2014), NCT02937844 (2016), NCT02866747 (2016), NCT02336165 (2015), NCT02337491 (2015), NCT03014804 (2017), NCT02550249 (2015).The preclinical data generated in the orthotopic glioma mice models suggests that combination treatment with PD1/PDL1 inhibitors and radiotherapy, natural killer cells, bevacizumab, and small-molecule inhibitors of apoptosis antagonists (SMCs) can successfully inhibit the tumors [11,12]. A blockage of PD1/PDL1 interaction restored antiglioma immunity and suppressed tumor growth in the Cl13 mouse glioma model with dysfunctional CD8 T cells due to chronic exposure to the tumor antigen and the high level of PD1 expression [13].

MLN4924, a pharmacological inhibitor of the NEDD8 E1 activation enzyme, is currently considered as a promising treatment for brain tumors [14-16]. MLN4924 can cross the blood-brain barrier and exhibits strong effectiveness towards tumors with an overactivated protein neddylation pathway, in vitro and in vivo [16,17]. The neddylation pathway is overactivated in gliomas and correlates with disease progression [17]. Treatment with MLN4924 results in tumor-specific cell cycle arrest, apoptosis and induction of the DNA damage response in the preclinical mouse glioma model [17,18].

The engagement of MLN4924 treatment with an anti-tumor immune axis is currently unexplored. Our work seeks to evaluate the impact of MLN4924 treatment on PDL1 expression on gliomas and on glioma cell immune evasion, in vitro. It was reported that treatment with MLN4924 is accompanied by a significant accumulation of the HIF1A transcriptional factor [19-22], which is a strong up-regulator of PDL1 expression. Potential HIF1A binding sites have been found in the PDL1 proximal promoter and the intron regions between the first and second, and the fourth and fifth exons of the PDL1 gene [23,24]. HIF1A-dependent PDL1 up-regulation was demonstrated in tumor-infiltrating myeloid-derived suppressor cells, in human breast and prostate cancer cells, in melanoma and mammary carcinoma cells, and in pulmonary pleomorphic and advanced oral squamous cell carcinomas [23-28].

In our manuscript, we confirm significant PDL1 overexpression in the clinical glioma samples, in the established and PDGx glioma cell lines, and in the CSF samples from tumor-bearing mice. By using several established glioma cell lines under a hypoxic condition mimicked by cell treatment with CoCl2, we verified HIF1A-dependent up-regulation of PDL1 in mRNA and protein levels. We evaluated two HIF1A binding domains in the intron region between the first and second exons of the PDL1 gene and confirmed accessibility and functionality of these domains in glioma cells by utilizing transcription initiation complexes guided by ghRNAs. We found that MLN4924 exhibited strong cytotoxicity against established and PDGx glioma cell lines, in vitro, and therefore, is a promising candidate for the treatment of brain tumors. However, we also observed a significant increase of HIF1A and PDL1 levels in all cell lines after treatment with MLN4924, which may lead to suppression of the immune response development, in vivo. We found that PDL1 up-regulation in glioma cells after MLN4924 treatment induced T-cell energy, which could be blocked by a PD1/PDL1 blockage. We conclude that using inhibitors of PDL1/ PD1 interaction with MLN4924 may improve the effectiveness of MLN4924 treatment, in vivo, via the reduction of immune-cell energy. We consider blocking the PD1/PDL1 pathway together with MLN4924 therapy as a potential strategy for glioma treatment; this strategy is in need of a detailed evaluation in preclinical orthotopic glioma mice models.

## Materials and Methods

### Cell culture and patient material

The U251 cell line was purchased from Sigma (Sigma-Aldrich, St. Louis, MO). The U87 and LN229 cell lines were purchased from the American Type Culture Collection (ATCC, Manassas, VA, USA). Note that although we utilized a commercial source of U87 cell line, this cell line is currently considered to lack a relationship with the primary tumor type of origin. The XD459 and JX10 primary patient-derived xenolines (PDGx) were previously established [29]. The GL261 cell line was a gift from Dr. King’s lab. The U251 cell lines with IDH-R132 mutations were developed and characterized in our recently published work [30]. The neuro spheres were formed and maintained in Neurobasal-A medium (Gibco, Carlsbad, CA, USA) supplemented with a B27 supplement without vitamin A (Gibco), N-2 supplement (Gibco), 2mM L-Glutamine (Media tech, Inc., Manassas, VA, USA), the basal growth factors (EGF, 20 ng/ml and bFGF, 20 ng/ml, were both purchased from Termo Fisher Scientifics (Grand Island, NY, USA)), and 100 U/ml Penicillin/Streptomycin (Media tech, Inc., Manassas, VA, USA). Whole blood samples were purchased from Hema Care (Hema Care Corporation, Van Nuys, CA, USA). PBMCs were separated by centrifugation in the Ficoll Hypaque density gradient. CD14 monocytes were depleted of PBMCs cell culture by adherence. Human CD3/CD28 Diamagnetic beads (Thermo Fisher Scientifics) and Il2 (Gibco) were used for stimulating primary T-cells. Before experiments, T-cells were loaded with cell-permeable green fluorescence dye, calcein AM, at 0.5-2 uM (Molecular Probes, Leiden, Netherlands) according to manufactory protocol for visualization.

### Immunohistochemistry

Standard immunohistochemistry protocol for paraffin embedded tissue section staining was used for the immunostaining of brain tumor samples (WHO I-IV) and control brain tissue. Immunostaining was performed in the UAB core facility. The Universal buffer consisting of 1% BSA, 0.2% non-fat powdered skim milk, 0.3% Triton X-100 and 1xPBS was utilized to prevent non-specific antibody binding. Immunostaining was performed overnight at 4°C with the Pdcd-1L1 (H-130) antibody from Santa Cruz Biotechnology at 1:200 dilution. Signal Stain Boost IHC Detection Reagent (HRP, rabbit) was used at room temperature for 30 min for signal detection, and a peroxidase-based substrate Kit (Vector Labs, Burlingame, CA, USA) was used for signal development. Harris hematoxylin solution (Fisher Scientific, Pittsburgh, PA, USA) was used for nuclear stains. VectaMount Mounting Medium (Vector Labs) was utilized for coverslip mounting. We define PDL1 immunostaining as strong if more than 50% of analyzed cells have a high PDL1 signal; medium, if 5% to 50% of analyzed cells have a high PDL1 signal; and low, if less than 5% of analyzed cells have a high PDL1 signal.

### Cloning

The pAC154-dual-dCas9VP160-sg expression plasmid was purchased from Addgene (Cambridge, Massachusetts, USA). The cloning of scrambled sgRNA and HIF1A sgRNA was performed with the following primers: Scramb-Bbs1-Forw 5’ CACCGGACGAGTCCTCTACAGCAC, Scramb- Bbs1-Ver 5’ AAACGTGCTGTAGAGGACTCGTCC, PDL1-HIF-Bbs1-A-Forw 5’ CACCGTTCGTGTTTTCCATAATTA, PDL1-HIF-Bbs1-A-Rev 5’ AAACTAATTATGGAAAA CACGAAC, PDL1-HIF-Bbs1-B-Forw 5’ CACCGCTCCTGTCTTATATATACGTG, PDL1-HIF-Bbs1-B-Rev 5’ AAACCACGTATATATAAGACAGGAGC. The cloning was verified by sequencing in the UAB core facility. DNA transfection was performed by using a Lonza SE cell line 4D-Nucleofector X Kit (Lonza, Koln, Germany, USA).

### Viability assay

Presto Blue cell viability reagent (Thermo Fisher Scientific) was used for a viability assay as previously described [30]. MLN4924 was added to the wells by using a multichannel pipette (XL 3000ITM, Denville, USA); each drug concentration was at least triplicated in the plate. mRNA collection and analysis by TaqMan technique. mRNA samples were collected and purified by using QIA shredder Kit (Qiagen, Germantown, MD, USA) and RNeasy Mini Kit (Qiagen). mRNA concentrations were evaluated by using the Nano Drop 1000 instrument (Thermo Scientific). mRNA samples were converted to cDNA by using Super Script iv Reverse Transcriptase (Invitrogen) and random primers (Thermo Fisher). The following inventory TaqMan probes were used: Hs01125301_m1 CD274, Mm03048248_m1 CD274, Hs00266705_g1 GAPDH, Hs99999901_s1 18S. The TaqMan PCR was performed on the 7900HT Fast Real-Time PCR System (Applied Biosystems).

### Collection and analysis of extracellular media

Cells were plated at 2×10^6^ per P10 plate. Fresh media (6 ml) was added to the cells and collected after 24 hours. Collected media was spun at 4°C, 5 min, 1200 rpm to remove any cells and cell debris. After debris removal, the protein content in the media was concentrated 6 to 8-fold by using the 10K protein concentrator PES (Thermo Scientific) at 4°C. The protein concentrate was reconstituted in Cell Lysis Buffer (Cell Signaling Technology) and analyzed by Western blot.

Antibodies, Reagents, and Drugs. HIF1A, Lamin A/C, hPDL1, anti-biotin HRP-linked, and anti-rabbit IgG1 HRP-linked antibodies were purchased from Cell Signaling Technologies (Danvers, MA, USA); Actin, HuR 3A2, PDL1, donkey anti-goat IgG-HRP, goat anti-mouse IgG1-HRP antibodies were from Santa Cruz Biotechnology (Dallas, Texas, USA); the mPDL1/B7-H1 antibody was from B&D systems (Minneapolis, MN, USA); the alpha Tubulin antibody was from Sigma (Sigma-Aldrich). Mouse PDL1 ELISA Kit was purchased from Boster (Pleasanton, CA, USA). MLN4924 was purchased from Active Biochem (Kowloon Bay, Hong Kong); the PD1/PDL1-inhibitor-1 was purchased from Cayman Chemical (Ann Arbor, Michigan, USA).

### Intracranial glioma model and collection of CSF

The intracranial injection of GL261 tumor cells was performed as previously described [29] in compliance with UAB animal care policy. The procedure of CFS collection was reported [31] and performed according to UAB animal care policy. The PDL1 level in CSF samples was analyzed by using the Mouse PDL1 ELISA Kit (Boster, Pleasanton, CA, USA). Mice were euthanized by using an IP injection of ketamine (500 mg/150 uL) in combination with an alpha 2-adrenergic receptor agonist followed by bilateral thoracotomy. The euthanasia method is approved by UAB Animal Care policy and by the AVMA Panel on Euthanasia, 2013 Edition.

### Statistical analysis

Statistical analysis and graphing were performed using Excel and Origin Pro software. Statistical significance was determined by Student’s t-test (to test for significant differences between two groups with equal or unequal variances) and was considered significant at p ≤ 0.05. Values are expressed as mean ± S.D. Statistically significant data is labeled by an asterisk in the graphs.

## Results

**Figure 1:**
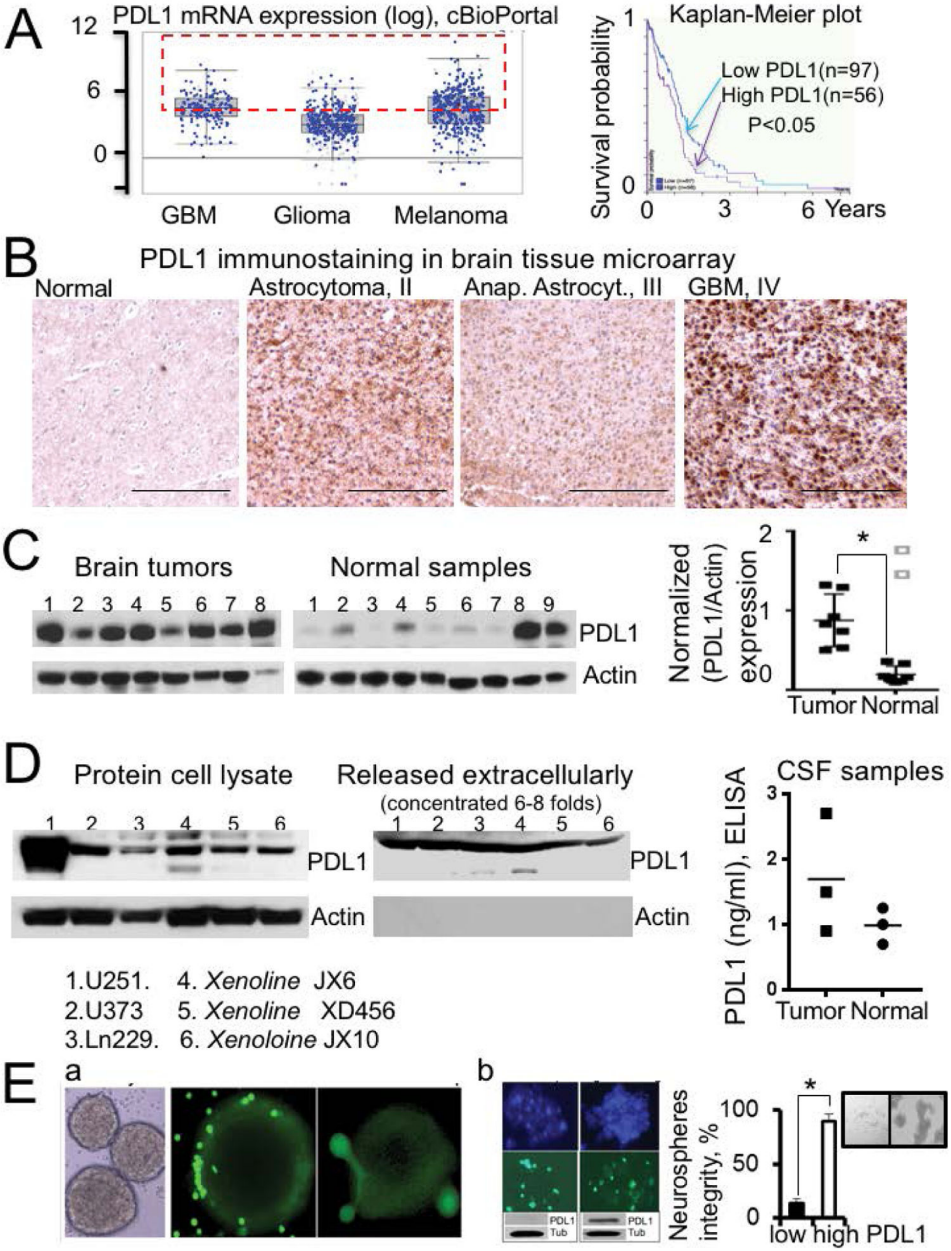
PDL1 expression in clinical brain tumor samples, glioma cell lines and CSF samples from the immunocompetent glioma mice model. **A)** Graph (left) represents PDL1 mRNA expression in brain tumors and melanomas (cBioPortal (http://www.cbioportal.org)). Note that the average PDL1 mRNA level in GBM is similar to the average PDL1 mRNA level in melanomas. The PDL1 values in GBM and low-grade gliomas which exceed the average PDL1 mRNA level in melanomas are highlighted in the red box. Kaplan-Meier plot (right) illustrates survival rates of glioma patients with high and low PDL1 expression levels; 9% versus 24% of 2 year survival for high and low PDL1 expression; the difference is significant, P=0.02 (data has been obtained from the Human Pathology Atlas). **B)** Immunohistochemical detection of PDL1 in the tissue microarray of normal and brain tumor samples. The images were taken at 40x magnification. **C)** Western blot illustrates PDL1 and Actin protein levels in control and brain tumor clinical samples. Graph represents PDL1 to Actin ratios for corresponding protein samples; 0.87 ± 0.32 (n=7) versus 0.19 ± 0.1 (n=7) for tumor and normal samples, respectively, the difference is significant, P=0.003. Two samples (marked by light grey color) of patients with hemorrhage have been excluded from the average. **D)** Western blots illustrate PDL1 protein levels in established and PDGx glioma cell lines (left) and in the extracellular media collected from these cell lines (right). The protein content of the collected media was concentrated six folds before an analysis (see method). The graph (right) provides PDL1 concentrations in CSF samples from control and tumor-bearing mice (see method). The immunocompetent glioma mice model with GL261 cells was utilized for this experiment. **E)** Images illustrate interactions of tumor neuro spheres from XD456 (a) and from two parental U251-IDH1-R132H cell lines with different PDL1 expression levels (b) with primary T-cells. Note that tumor neuro spheres with high PDL1 levels keep their integrity after 48 hours of interaction with primary T-cells (illustrated in the insert). The graph represents the average percent of neuro spheres after 48 hours of interaction with T-cells, 89 ± 7% (n=4) and 14 ± 5% (n=4) for cell lines with high and low PDL1 levels, respectively. The difference is significant, P=0.0002. Primary T-cells were loaded with calcein, AM (green) before experiments for visualization.

PDL1 expression in clinical brain tumor samples, glioma cell lines and CSF samples from the glioma mice model. PDL1 expression in clinical glioma samples has drawn huge attention in the last couple of years and is still controversial. First, we compared PDL1 mRNA expression in low and high-grade gliomas to PDL1 expression in melanomas by utilizing the cBioPortal portal for cancer genomics (http://www.cbioportal.org). We found that the average levels of PDL1 mRNA in GBM and melanomas are similar (Figure 1); the average PDL1 mRNA level in low-grade gliomas is three times lower than those in GBM and melanomas. Although low-grade gliomas exhibited an overall decrease of PDL1 expression, about 20-30% of low-grade gliomas (with verified IDH1-R132 single allele mutation) demonstrated PDL1 values exceeding the average PDL1 level in melanomas (Figure 1A). Our data from clinical glioma samples confirmed high heterogeneity of PDL1 expression with a range from 0.3 to 4.2 for PDL1/GAPDH mRNA ratio. 60% of analyzed samples showed PDL1/GAPDH mRNA ratios higher than the average control (0.58 ± 0.2, n=4), the difference between the average PDL1/GAPDH ratios of control and tumor samples was not significant, p=0.2. According to current data provided in the Human Pathology Atlas, PDL1 overexpression is associated with a significant shortening of patient survival. A Kaplan-Meier plot of glioma patient survival with high and low PDL1 levels is presented in Figure 1; the difference is significant, p=0.02. Next, we analyzed PDL1 expression on the protein level. Figure 1B illustrates PDL1 immunostaining in a brain tumor tissue array. We found PDL1 immunostaining strong in 29%, medium in 36%, and weak in 35% of low-grade glioma samples (fourteen samples total). GBM samples exhibited strong PDL1 immunostaining in 33%, medium in 50% (3 of 6), and weak in 17% of samples (six samples total). An analysis of PDL1 expression in the clinical samples by Western blot confirmed significant up-regulation of PDL1 expression in GBM compared to normal tissue (Figure 1C); the PDL1/Actin ratios for corresponding samples are presented in the graph in Figure 1-right (0.87 ± 0.32 (n=7) versus 0.19 ± 0.1 (n=7) for tumor and normal samples, respectively, p=0.003).

We detected substantial PDL1 protein expression in all evaluated PDGx and established glioma cell lines by Western blot (Figure 1D, left). Also, we were able to detect the PDL1 protein in extracellular media collected from corresponding PDGx and established glioma cell lines (Figure 1D, right) (see method). The analysis of CSF samples obtained from normal and tumor-bearing mice (immunocompetent glioma mice model with GL261 cells) by puncturing the cisterna magna revealed that PDL1 is present in normal samples at a concentration of around 1 ng/ml and increases to up to 2.8 ng/ml in mice with tumors (Figure 1D, graph) (see methods for technique of CSF collection and analysis). We anticipate that the up-regulation of PDL1 in gliomas may protect gliomas from T-cell based immunotherapy and contribute to induction of T cell hyperresponsiveness. We formed tumor neurospheres from PDGx and established glioma cell lines and confirmed interactions between allogenic T cells (loaded with cell-permeable, green fluorescence dye calcein, AM for visualization) and tumor neurospheres (Figure 1E(a)) for several cell lines. We formed neuro spheres from parental U251-IDH-R132H glioma clones characterized in our recent manuscript [30], with low and high PDL1 expression levels (at least 10 folds difference in PDL1 expression levels) and evaluated the outcome of tumor neuro spheres’ encounters with primary T cells (depleted from CD14 monocytes and stimulated with CD3/CD28 Diamagnetic beads). We found that neuro spheres with high PDL levels keep their integrity after an encounter with T cells and, thus, stay resistant to T cell treatment (Figure 1E(b)). After 48 hours of interaction with T cells, an average of 89 ± 7% (n=4) neurospheres with high PDL1 levels keep their integrity versus 14 ± 5% (n=4) neuro spheres with low PDL1 levels (Figure 1E(b)); the difference is significant, P=0.0002. In the following experiment, we co-incubated primary T cells with media from XD456 and U251 glioma cell lines with and without blockage of PDL1/PD1 interaction by PDL1-inhibitor-1 (4 uM). In agreement with other researchers, we confirmed that the blockage of PD1/PDL1 interaction shifted the steady-state profile of T cells to a more proliferative one (on 28 ± 12%, n=3 and 25 ± 8%, n=3 during 24 hours of co-incubation with media from XD456 and U251 cell lines, respectively). Thus, our data confirmed that PDL1 is up-regulated in gliomas and may protect tumors from T-cell based immunity.

**Figure 2:**
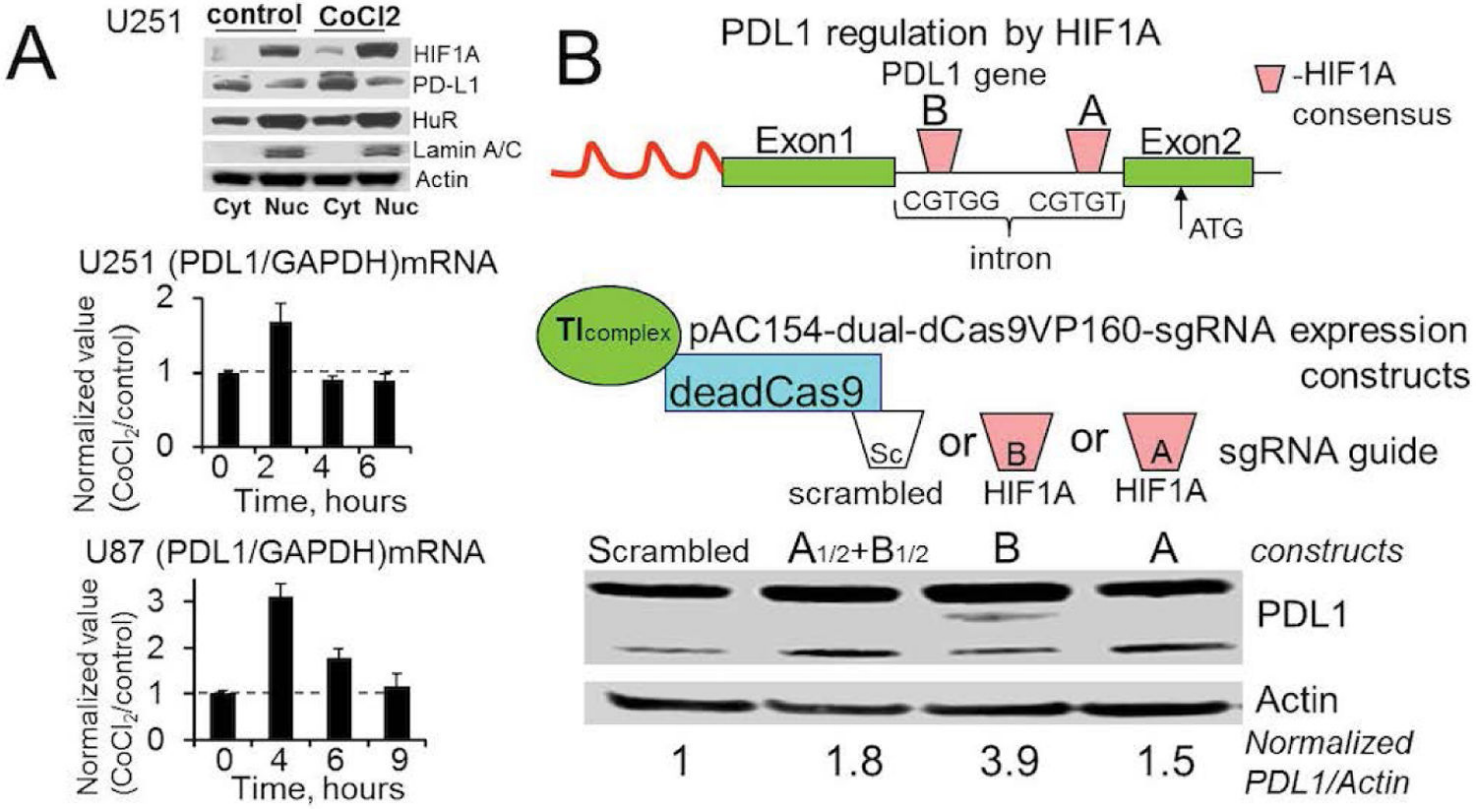
HIF1A accumulation evokes PDL1 up-regulation in glioma cell lines. **A)** HIF1A accumulations induced by CoCl2 treatment of U251 and U87 cell lines evoke PDL1 up-regulation in protein and mRNA levels. Western blot illustrates HIF1A and PDL1 levels in nuclear and cytoplasmic fractions, respectively, in control and after treatment with CoCl2 (85uM). Lamin A/C and alpha Tubulin were utilized to verify nuclear and cytoplasmic fractions, respectively. Graphs illustrate normalized PDL1/GAPDH mRNA ratios after cell treatment with CoCl2 (85uM) at different time points. In each experiment, data has been normalized to the corresponding ratios in untreated cells, results are presented as mean ± S.D. **B)** Transcription initiation complexes encoded by pAC154-dual-dCas9VP160 plasmids, guided by sgRNAs to the HIF1A binding domains in the first intron of the PDL1 gene, evoke PDL1 overexpression. Western blot illustrates PDL1 and Actin protein levels in U251 cells after transfection with plasmids encoding control (scrambled sequence) sgRNA or HIF1A sgRNAs. Note that transcription initiation complexes guided by sgRNAs to the HIF1A binding domains (A), (B), and (A) with (B) simultaneously increased PDL1 expression by 1.5, 3.9 and 1.8 folds, respectively, compared to the control (PDL1 expression in the presence of transcription initiation complexes guided by scrambled sgRNA). In each experiment, PDL1 expression was normalized to the Actin expression.

**Figure 3:**
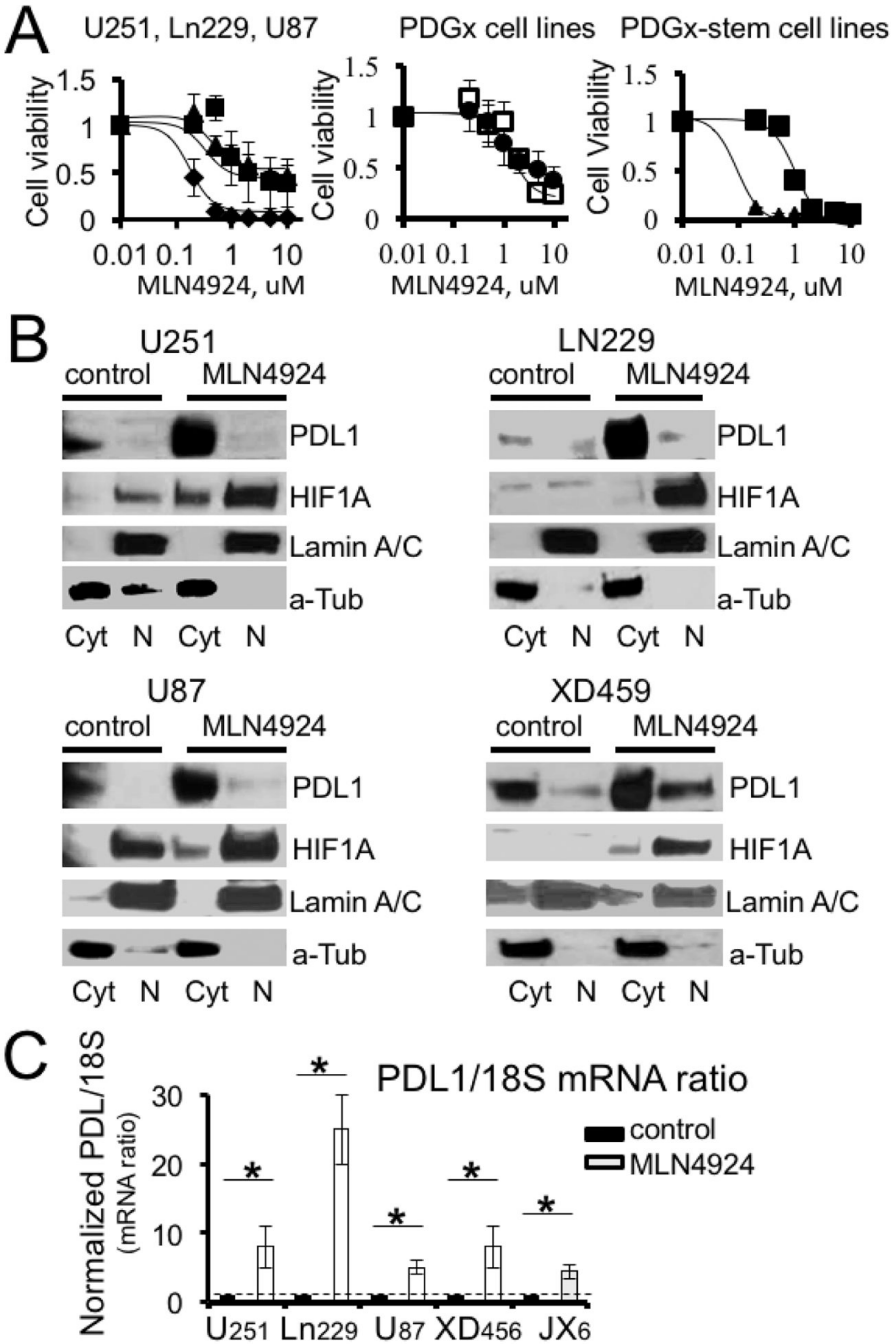
MLN4924 treatment induces up-regulation of HIF1A and PDL1 in glioma cell lines. **A)** The inhibitory dose- response curves for MLN4924 in established, PDGx and PDGX-stem glioma cell lines. The IC50s are 0.3 ± 0.2 uM (n=4), 2.7 ± 1 uM (n=6), 3 ± 2 uM (n=3), 3 ± 1 uM (n=4), 2.9 ± 0.5 uM (n=4), 0.8 ± 0.2 uM (n =4), 0.2 ± 0.1uM (n=4) for LN221, U251, U87, XD456, JX10, XD456-stem, X14P-stem cell lines, respectively, after treatment with MLN4924 for 5 days. **B)** Western blots illustrate HIF1A and PDL1 protein levels in nuclear and cytoplasmic fractions in the control and after treatment with MLN4924 (1 uM, 5 days). LaminA/C and alpha Tubulin were utilized to verify nuclear and cytoplasmic fractions, respectively. **C)** The graph illustrates normalized PDL1/18S mRNA ratios after treatment with MLN4924 (1uM, 5 days) for different cell lines. Note the significant enhancement of the PDL1/18 mRNA ratio for all cell lines after MLN4924 treatment: 8 ± 3, 25 ± 5, 5 ± 1, 8 ± 3, 4.5 ± fold increase compared to the corresponding control values for U251, Ln229, U87, XD456, JX6 cell lines, respectively, P<0.05, n=3.

HIF1A accumulation leads to PDL1 up-regulation in glioma cell lines. HIF1A/PDL1 axis plays a significant role in PDL1 up-regulation in different types of cancer. To evaluate HIF1A/PDL1 signaling axis in gliomas, we used U251 and U87 established glioma cell lines. The hypoxic HIF1A accumulation was mimicked by cell treatment with CoCl2, which blocks HIF1A degradation. We observed a significant enhancement of PDL1 mRNA levels in both cell lines after treatment with CoCl2 (Figure 2A). The maximum PDL1 mRNA levels were detected after 2 and 4 hours of treatment with CoCl2 for U251 (1.68 ± 0.25, n=3 increase) and U87 (3.1 ± 0.28, n=3 increase) cell lines, respectively. The PDL1 mRNA values were normalized to the corresponding GAPDH mRNA values in each experiment. The translation initiation complexes encoded by pAC154-dual-dCas9VP160 plasmids and guided by sgRNA to two HIF1A binding domains in the first intron of the PDL1 gene (Figure 2B-top) enhanced PDL1 protein expression by 1.5 ± 0.3, n=3 and 3.5 ± 0.6, n=3 fold for sites A and B, respectively, compared to PDL1 expression after cell transfection with translation initiated complexes guided by scrambled sgRNA (as a control) (Figure 2B). The simultaneous stimulation of both HIF1A binding sites increased PDL1 expression by 1.6 ± 0.3, n=3 fold compared to the control (Figure 2B). Our data confirmed the HIF1A/PDL1 signaling axis in glioma cell lines and the accessibility and functionality of at least two HIF1A binding sites in the PDL1 gene. MLN4924 treatment induces PDL1overexpression in glioma cell lines. We predict that HIF1A/PDL1 axis may be overactivated in glioma cells after treatment with MLN4924. Figure 3A illustrates MLN4924 inhibitory dose response curves for PDGx, established, and PDGx-stem human glioma cell lines, in vitro. The IC50s were 0.3 ± 0.2 uM (n=4), 2.7 ± 1 uM (n=6), 3 ± 2 uM (n=3), 3 ± 1 uM (n=4), 2.9 ± 0.5 uM (n=4), 0.8 ± 0.2 uM (n=4), 0.2 ± 0.1 uM (n=4) for LN221, U251, U87, XD451, JX10, XD45-stem, X14P-stem cell lines, respectively, after treatment with MLN4924 for 5 days. Note a remarkable loss of cell viability after treatment with MLN4924; however, we also confirmed a significant enhancement of HIF1A protein levels in all evaluated PDGx and established glioma cell lines after treatment with MLN4924, 1 uM for 5 days (Figure 3B). As was expected, HIF1A accumulation was accompanied by a significant increase of PDL1 in mRNA and protein levels (Figures 3B and 3C). The average enhancements of PDL1/18S mRNA ratio after MLN4924 treatment compared to untreated cells were 8 ± 3, 25 ± 5, 5 ± 1, 8 ± 3, 4.5± 1 folds for U251, Ln229, U87, XD456, JX6 cell lines, respectively, based on three experiments (Figure 3C).

We predict that MLN4924-dependent PDL1 up-regulation in glioma cells may enhance T-cell energy during a T-cell encounter with glioma cells treated with MLN4924. To evaluate our hypothesis, we performed a comparison of the interaction of allogenic T-cells preactivated by CD3/CD28 beads with:

a) Glioma cells (U251 and XD456 cell lines) alone,
b) Glioma cells plus a PD1/PDL1 blockage,
c) Glioma cells treated with MLN4924,
d) Glioma cells treated with MLN4924 plus a PD1/PDL1 blockage.

**Figure 4:**
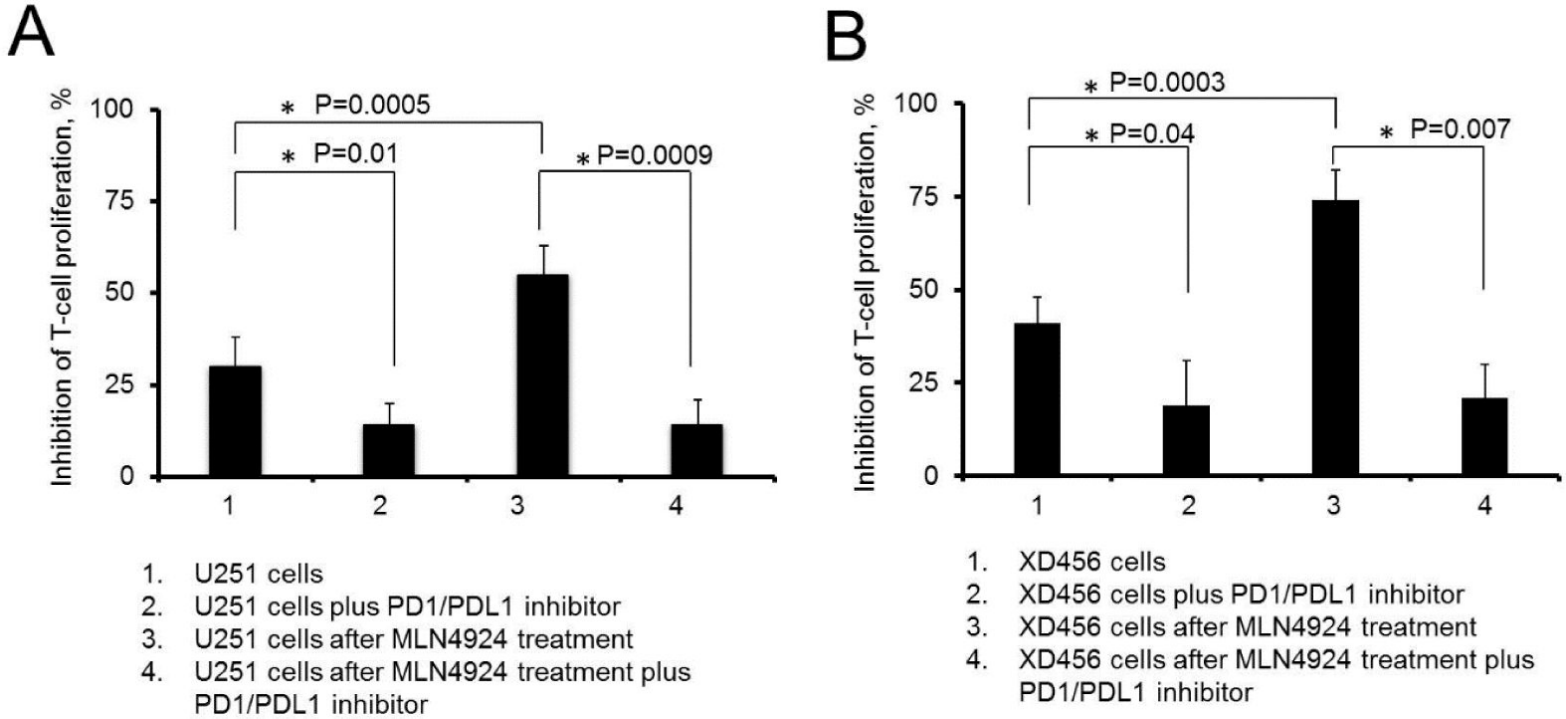
Glioma cells treated with MLN4924 decrease T-cell proliferation via utilization of PD1/PDL1 signaling pathway. **A)** The graph illustrates an inhibition of T-cell proliferation after T-cell encounter with U251 cells (1); with U251 cells in the presence of an inhibitor of PD1/PDL1 interaction (2); with U251 cells treated with MLN4924 (1uM, for 4 days) (3); with U251 cells treated with MLN4924 (1uM, for 4 days) and in the presence of an inhibitor of PD1/PDL1 interaction (4). After MLN4924 treatment, glioma cells were washed and placed in the media with/and without an inhibitor of PD1/PDL1 interaction (4 uM). Note, that glioma cells treated with MLN4924 induce a stronger decrease of T-cell proliferation compared to untreated cells (55 ± 8% (n=4) versus 30 ± 8% (n=4), respectively, the difference is significant with P=0.0005). The reduction of T-cell proliferation induced by glioma cells treated with MLN4924 is inhibited in the presence of an inhibitor of PD1/PDL1 interaction (P=0.0009, n=4). **B)** The graph illustrates an inhibition of T-cell proliferation after T-cell encounter with XD456 cells (1); with UXD456 cells in the presence of an inhibitor of PD1/PDL1 interaction (2); with XD456 cells treated with MLN4924 (1uM, for 4 days) (3); with XD456 cells treated with MLN4924 (1uM, for 4 days) and in the presence of an inhibitor of PD1/PDL1 interaction (4). After MLN4924 treatment, glioma cells were washed and placed in media with/and without an inhibitor of PD1/PDL1 interaction. Note, that glioma cells treated with MLN4924 induce a stronger decrease of T-cell proliferation compared to untreated cells (74 ± 8% (n=4) versus 41 ± 7% (n=4), respectively, the difference is significant with P=0.0003). The reduction of T-cell proliferation induced by glioma cells treated with MLN4924 is inhibited in the presence of an inhibitor of PD1/PDL1 interaction (P=0.007, n=4).

After MLN4924 treatment (1 uM, four days), glioma cells were washed and placed in the media with/and without an inhibitor of PD1/PDL1 interaction (4 uM). T-cell proliferation was analyzed 48 hours after co-incubation with glioma cells. The results of these experiments are summarized in Figure 4. The glioma cells after MLN4924 treatment have stronger potential to induce T-cell anergy compared to untreated glioma cells (the average decreases in T-cell proliferation were stronger by 24 ± 3% (n=4, P=0.0005) and 32 ± 3% (n=4, P=0.0003) after encounters with MLN4924 treated U251 and XD456 glioma cells, respectively, compared to T-cell proliferation after an encounter with untreated U251 and XD456 cells). Importantly, the enhancement of T-cell anergy induced by glioma cells treated with MLN4924 was inhibited in the presence of PD1/PDL1 inhibitor-#1 (Figure 4), suggesting that PD1/PDL1 interaction is the main signaling path involved in T-cell anergy after glioma cell treatment with MLN4924. We conclude that the co-utilization of a PD1/PDL1 blockage with MLN4924 treatment reduces PD1/PDL1-dependent immune-cell energy associated with MLN4924-dependent PDL1 up-regulation. Thus, we recommend using inhibitors of PDL1/PD1 interaction with MLN4924 treatment to improve anti-tumor immunity and to reduce glioma progression.

## Discussion

The brain tumor is the most devastating and incurable disease of the 21st century; it is characterized by high tissue heterogeneity and undergoing a fast transformation from low-grade I-II to high- grade III-IV malignancy. According to the analysis and projection of the national cost of cancer care for 2010-2020, brain cancer is the most expensive in terms of the annual net cost of care per patient and is ranked third for lost productivity due to cancer deaths among all adults with cancer [32]. 90% of patients with high-grade gliomas experience tumor recurrence despite maximum surgical tumor resections, radio, and chemotherapies [33,34]. The overall median of patient survival is 15-18 months, and only about 10% of patients stay alive after five years. We believe that a new multimodality therapy, synergistically targeting the intrinsic axis of tumor cell survival and promoting anti-tumor immunity for long-term treatment, may significantly improve glioma patient survival.

Our manuscript emphasizes that MLN4924, in combination with the blockage of PD1/PD1 interaction, may be a potential strategy for glioma treatment. First, MLN4924 exhibits strong cytotoxicity towards glioma cell lines and crosses the blood-brain barrier. Second, MLN4924 has a minimal impact on the intrinsic axis of immune cell growth compared to most of anti-cancer chemotherapeutics, which completely wipe out all types of proliferating cells, including immune cells. The reported effects of MLN4924 on the immune system consist of:

a) An increase of CD4-induced epitope exposure in cells infected with HIV-1 viruses, however, without significant alteration of host-initiated antibody-dependent cellular cytotoxicity [35],
b) A partial suppression of graft-versus-host disease immunepathologies [36,37],
c) A partial decrease of airway inflammatory responses [38]. In our manuscript, we demonstrate significant up-regulation of the key immunosuppressive checkpoint molecule, PDL1, in glioma cells after treatment with MLN4924, and thus, PDL1-dependent T-cell energy after an encounter with glioma cells. In agreement with our observation, the capacity of a PD1/PDL1-pathway blockage to enhance CD4 and CD8 T-cell responses and moreover, to improve anti-BTLA or anti-TIM3 therapy during allogeneic T and DC cell interactions have been recently confirmed [39].

## Conclusion

This study extended our knowledge of PDL1 regulation in gliomas, the microenvironment of gliomas, and after glioma treatment with MLN4924. Therefore, we suggest that a blockage of PD1/PDL1 interaction during/or after MLN4924 treatment may significantly improve the efficiency of MLN4924 therapy via the reduction of PD1/PDL- dependent immune cell energy and the promotion of anti-tumor immunity. Our data justifies that PDL1 up-regulation in gliomas and the glioma microenvironment is an important chemotherapeutic target and is in agreement with other manuscripts [40-44].
